# Sonographic Mimic of Ovarian Ectopic Pregnancy by Point-of-Care Ultrasound

**DOI:** 10.7759/cureus.72830

**Published:** 2024-11-01

**Authors:** Michael Boniface, Aaron Kline, Taylor Zeglam, Giuliano DePortu

**Affiliations:** 1 Emergency Medicine, Mayo Clinic, Jacksonville, USA; 2 Radiology, University of Florida College of Medicine, Gainesville, USA; 3 Emergency Medicine, University of Florida College of Medicine, Gainesville, USA

**Keywords:** ectopic pregnancy, emergency, fast exam, graafian follicle, point-of-care ultrasound

## Abstract

The detection of ectopic pregnancy (EP) by point-of-care ultrasound has become an integral competency of emergency medicine practice. Clinical algorithms usually include a female of childbearing age, positive urine or serum human chorionic gonadotropin (hCG) test, and an ultrasound evaluation to assess the presence or absence of intrauterine pregnancy. This case report illustrates incidental findings observed during focused assessment with sonography in trauma (FAST examination), which initially suggested an intra-ovarian EP. The clinical images and interpretation are subsequently discussed. This case is important to the practice of general emergency medicine in that it illustrates the importance of contextual interpretation, management, and recognition of normal anatomical features and incidental findings commonly encountered during point-of-care sonography.

## Introduction

Ectopic pregnancy (EP) is defined as the extra-uterine implantation of a fertilized embryo and occurs in up to 1.4-2% of all natural conceptions [1]. However, the incidence may be as high as 7.5% in the population of all women presenting to United States emergency departments with a positive pregnancy test [2]. Risk factors for EP include prior infections such as pelvic inflammatory disease, previous EP, endometriosis, and previous abdominal or pelvic surgeries. While the vast majority of EPs occur in the fallopian tubes, up to 3.2% have been observed in an ovarian location [3]. Ovarian EP can be particularly challenging as patients with rupture are much more likely to present in hemodynamic shock at the time of diagnosis, experience larger volume intraoperative blood loss, and require prolonged hospitalization [4]. Although a high index of suspicion and prompt evaluation can reduce poor outcomes, a lack of definitive management can lead to devastating results. Up to 9% of all maternal deaths are due to EP [5].

## Case presentation

**Figure 1:**
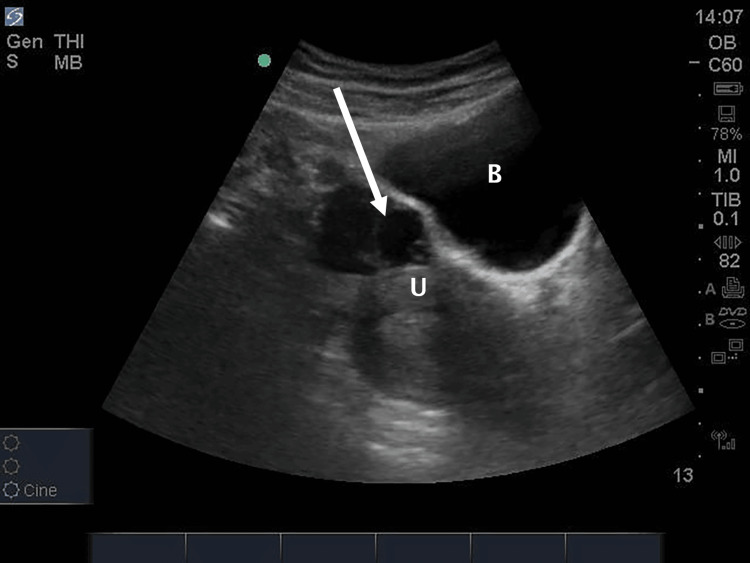
Transabdominal ultrasound exam showing a sagittal view of the pelvis. An anechoic cystic structure is seen within the left ovary with an additional internal ring-like structure, initially suspected to represent a yolk sac (arrow). The fundus of the uterus (U) and bladder (B) are also visualized.

**Figure 2:**
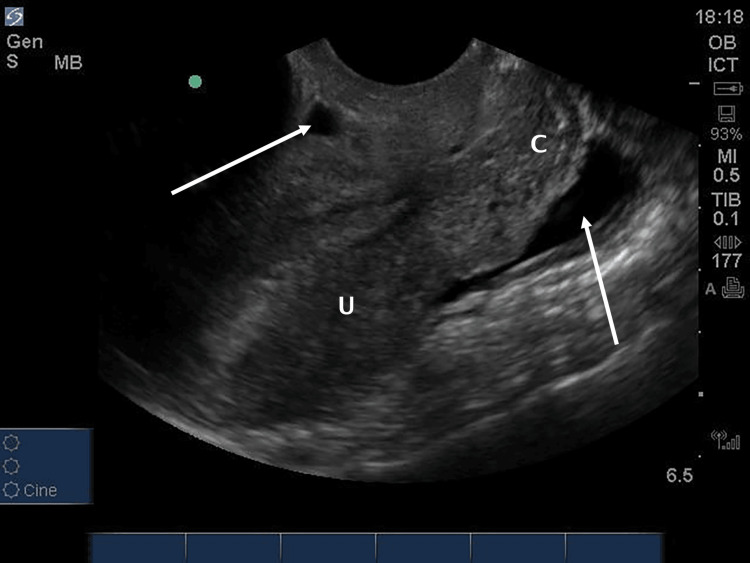
Endovaginal sagittal view of the cervix (C) and uterus (U) demonstrates anechoic free fluid in the retrouterine pouch (pouch of Douglas) and a small amount of free fluid anterior to the uterus arrows).

**Figure 3:**
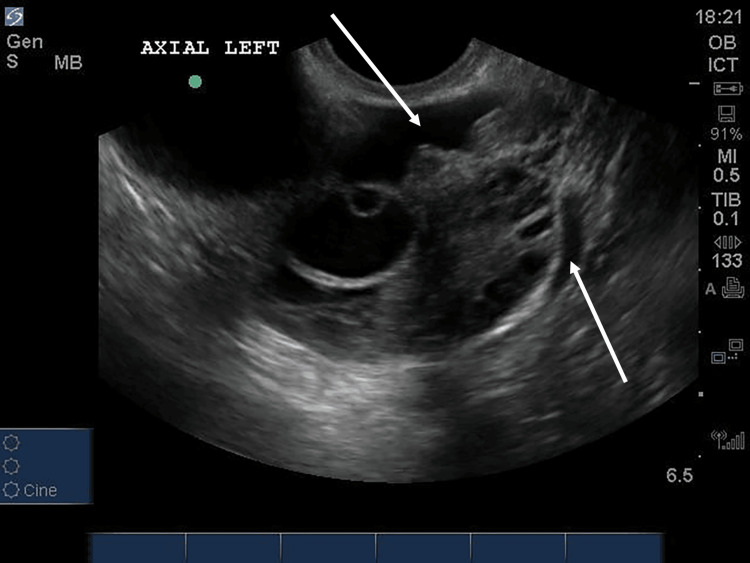
Transverse view of the left ovary demonstrates anechoic free adnexal fluid (arrows). In the left ovary, an anechoic cyst within a cyst is again visualized.

A 34-year-old woman presented to the emergency department following a low-energy mechanism motor vehicle collision for which she was the restrained driver. Her primary complaint was mild diffuse lower abdominal discomfort. Her past medical history was unremarkable and she was not on any systemic anticoagulation. On physical examination, she was noted to be tachycardic on arrival with some tenderness to palpation in the suprapubic region of the abdomen. The FAST examination was conducted as part of her secondary survey. In the suprapubic window, free fluid was demonstrated in the pelvis. An incidental cystic lesion was identified within the left ovary (Figure 1). The patient endorsed having no menstrual period for many months since she last gave birth and denied any contraceptive use in the interim. A subsequent endovaginal ultrasound exam was performed by the emergency physician while awaiting urine hCG results to evaluate for clinical suspicion of ruptured ovarian EP. The endovaginal ultrasound characterized small free pelvic fluid in the pouch of Douglas with trace fluid anterior to the uterus (Figure 2) and fluid in the left adnexa adjacent to the ovary (Figure 3). Within the left ovary, an anechoic cyst within a cyst was visualized. This was initially suspected to represent a yolk sac within a gestational sac by the emergency medicine physician. However, urine hCG and quantitative serum hCG were undetectable. The remainder of her diagnostic evaluation was unremarkable. There were no identified traumatic injuries requiring intervention. The patient was ultimately discharged home from the ED in good condition.

## Discussion

The sonographic mimic of EP detailed in the figures above represents a mature Graafian (tertiary) follicle. The mature ovarian follicle appears in the pre-ovulatory phase, just prior to oocyte expulsion from the follicle. The follicle was mistaken for an intraovarian gestational sac and the cumulus oophorus was mistaken for a yolk sac. These factors misled the emergency medicine physician to the incorrect postulation of primary ovarian EP. As quantitative serum beta-hCG was undetectable, the diagnosis of EP was subsequently excluded.

**Figure 4:**
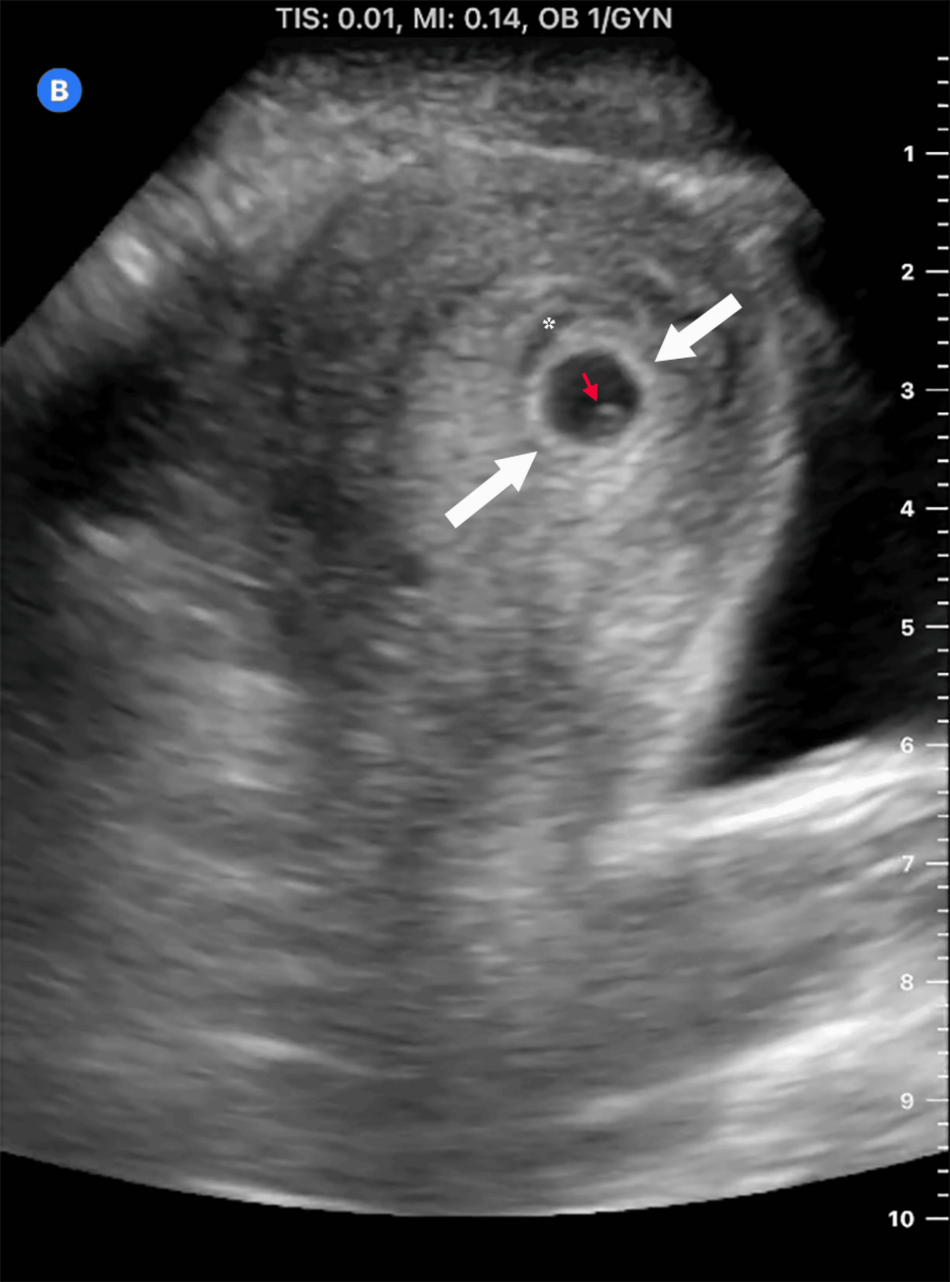
A true intrauterine gestational sac containing a visible yolk sac at approximately six weeks gestation. White arrows: bound gestational sac with thick hyperechoic walls. Red arrow: yolk sac. Asterisk: incidental small subchorionic hemorrhage.

Primary ovarian EP is an exceedingly rare phenomenon, representing up to 3.2% of all EPs [3]. There are sonographic features that can help distinguish a true EP from a normal ovarian follicle. The border of the ovarian cyst in this patient is thin-walled. In addition, the cumulus oophorus, which is a layer of thickened zona granulosa cells, will always be located on the periphery of the mature follicle. The appearance of a true gestational sac, by comparison, is that of a wide hyperechoic wall surrounding an internal anechoic space [6]. An example showing the typical appearance of a true gestational sac (from a different patient) is shown in Figure 4. Oftentimes, the ovarian EP will appear like a complex and heterogeneous cystic structure and cannot be differentiated from a hemorrhagic ovarian cyst on ultrasound [7-8]. Rarely, fetal tissue or cardiac activity may be visualized in an ovarian EP [2]. Other signs that should raise suspicion for a true EP include large volume or heterogeneous free fluid.

The FAST examination has become the standard of care in the evaluation of patients with blunt abdominal trauma and has excellent test characteristics for the detection of free peritoneal fluid including a sensitivity between 73% and 88%, specificity between 98% and 100%, and accuracy of 96-98% [9]. However, the observation of free pelvic fluid in women of reproductive age should be interpreted in the appropriate clinical context and correlated with other physical findings and index of suspicion. Physiologic free fluid may be observed commonly in the posterior cul-de-sac on ultrasound as a normal finding, independent of trauma or other pathological processes [10]. 

## Conclusions

Physiologic-free pelvic fluid is commonly observed in women of ovulatory age and is seldom of clinical consequence. Ovarian EP is rare but is more likely to present with hemorrhagic shock and worsened clinical outcomes. An astute emergency medicine provider should retain a high index of suspicion for conditions including blunt abdominal trauma and ruptured EP, as they represent pathologies associated with high morbidity and mortality. However, patients must also be evaluated in the appropriate clinical context, with careful consideration given to sonographic mimics and incidental findings that may inappropriately guide therapy.

## References

[1] Chow WH, Daling JR, Cates W Jr, Greenberg RS (1987). Epidemiology of ectopic pregnancy. Epidemiol Rev.

[2] Stovall T, Kellerman A, Ling F (1990). Emergency department diagnosis of ectopic pregnancy. Ann Emerg Med.

[3] Bouyer J, Coste J, Fernandez H, Pouly JL, Job-Spira N (2002). Sites of ectopic pregnancy: a 10 year population-based study of 1800 cases. Hum Reprod.

[4] Joseph RJ, Irvine LM (2012). Ovarian ectopic pregnancy: aetiology, diagnosis, and challenges in surgical management. J Obstet Gynaecol.

[5] Chang J, Elam-Evans LD, Berg CJ (2003). Pregnancy related mortally surveillance — United States, 1991-1999. MMWR Surveillance Summaries.

[6] Comstock C, Huston K, Lee W (2005). The ultrasonographic appearance of ovarian ectopic pregnancies. Obstet Gynecol.

[7] Nwanodi O, Khulpateea N (2006). The preoperative diagnosis of primary ovarian pregnancy. J Natl Med Assoc.

[8] Begum J, Pallavee P, Samal S (2015). Diagnostic dilemma in ovarian pregnancy: a case series. J Clin Diagn Res.

[9] Hoff WS, Holevar M, Nagy KK (2002). Practice management guidelines for the evaluation of blunt abdominal trauma: the East practice management guidelines work group. J Trauma.

[10] Sirlin CB, Casola G, Brown MA, Patel N, Bendavid EJ, Deutsch R, Hoyt DB (2001). Us of blunt abdominal trauma: importance of free pelvic fluid in women of reproductive age. Radiology.

